# Fracture Mode Transition during Assembly of TC4 High-Lock Bolt under Tensile Load: A Combined Experimental Study and Finite Element Analysis

**DOI:** 10.3390/ma15124049

**Published:** 2022-06-07

**Authors:** Derong Feng, Chenxi Dong, Yunpeng Hu, Yamei Wang, Jianhua Ma, Zhangdong Huang, Qiang Wan

**Affiliations:** 1 Henan Key Laboratory of Fastening Connection Technology, Xinyang 464000, China; xyfdrong@126.com (D.F.); dongchenxi2008@163.com (C.D.); hyp4115211@163.com (Y.H.); 23hewym@163.com (Y.W.); majianhua0402@163.com (J.M.); 2 Henan Aerospace Precision Machining Co., Ltd., Xinyang 464000, China; 3 College of Engineering, Huazhong Agricultural University, Wuhan 430070, China; wyzhangdong@126.com

**Keywords:** high-lock bolt, tensile test, FEA, preload, fracture mode

## Abstract

Fracture during the assembly process is an important failure mode for high-lock bolts used in the aviation industry, which greatly increases the potential of unpredictable accidents during service. In the current study, the underlying reasons for fracture during the assembly of a TC4 high-lock bolt was investigated using a tensile test and finite element analysis (FEA). The microstructure of the as-received bolt consisted of a high proportion of α phase, some β phase, and a small amount of α′ phase formed via martensite phase transformation during the rammer process. The experimental force–displacement curves revealed an average yield load of 55.9 kN and a breaking load of 67.65 kN. The corresponding yield strength was calculated to be 0.9 GPa, which was smaller than the standard value of TC4. This was attributed to the preload-induced stress concentration on the thread surface, leading to obvious strain hardening, which can lead to crack initiation. The effect of preload was further confirmed by the fractographies in which the initial crack was observed on the thread surface. The fractographies suggested that hybrid fracture occurred on the tensile loaded bolt. The initial failure was brittle fracture on the thread surface, transforming into ductile fracture in the screw. The results can contribute to understanding the effect of preload on the load carry capacity of high-lock bolts and provide a strategy to design its assembly specification.

## 1. Introduction

Recently, with the development of the Chinese aviation industry, there has been an increasing demand for high-quality aerospace fasteners [1]. Basically, aerospace fasteners require high strength, low weight, and excellent corrosion resistance [2]. Since titanium alloy fasteners have been successfully used in aircraft to reduce weight since the 1950s, they have drawn the attention of the aviation industry and been widely applied in many different aircraft in China [3]. TC4 (Ti-6Al-4V) high-lock bolts with a built-in preload and self-locking is one of the most commonly used connectors among titanium fasteners [4]. However, many fracture cases have been reported even when the applied load is lower than the strength of TC4 [5]. This is supposedly related to the assembly process.

To explore the underlying mechanism of fracture during assembly, there are three main traditional approaches. The first approach is an analysis of the microstructure and mechanical properties of the fracture surface in the failure bolt. Indeed, this method has been widely applied in bolt fracture. Guo et al. studied the fracture behaviors of 42CrMo via macroscopic and microscopic observations and mechanical property testing, suggesting that structure defects such as sulfide inclusions could greatly decrease the fatigue strength and yield strength by providing easy access to crack growth [6]. Zhang et al. also presented a detailed fractographic study and metallurgical analysis, which revealed that fatigue fracture was the main failure mechanism of bolts assembled on fans used in internal combustion engines according to the observation of micro-cracks in the thread tip [7]. The results successfully revealed the fracture causes of TC4 bolts and provided usage specifications. However, it is still difficult to detect the load carry capacity of bolt threads using this method [8,9]. The second approach is an experimental simulation using tensile tests and shear tests to recreate the load carry conditions of fasteners during assembly and service. Through this method, the true stress and strain value of the bolt under different types of forces can be obtained [10]. Moreover, the detailed stress distribution of the bolt during mechanical test should be studied to illustrate the mechanisms underlying fracture. This can only be achieved using the third approach, finite element analysis (FEA) [11]. FEA allows building a model to simulate the processing or service conditions to obtain the details of stress distribution and reveal the mechanisms underlying fracture [12,13].

Tensile experimental strategies are widely used in combination with FEA to reveal the detailed fracture evolution [14,15]. The technique exhibits a mutual promotional relationship; the accuracy of the finite element model can be corrected by experimental results, while providing a more reliable detailed stress distribution. Zhang et al. analyzed the fracture of a twin-roll press using the abovementioned method and found that the failure mode was fatigue fracture induced by stress concentration resulting from an unreasonable structural design [16]. Analyses of experimental and predicted SBCSOF shapes revealed the mechanism for the shear fracture under combined shear and compressive bending deformations [17]. For bolts, a similar method also was used to detect the usage standard of bolts in different situations [18,19]. However, most reported bolt fractures occur in the bolt bar, and the finite element model always simplifies the bolt as a bar with a fixed diameter. The thread was ignored in most previous studies, leading to a lack of understanding of thread failure, especially the effect of thread damage on the bolt fracture [20]. Indeed, the fracture of high-lock bolts during assembly mostly occurs in the thread because of the large pre-tightening force and its small size. However, most thread-related numerical studies mainly focused on the loosening behavior instead of the preload-induced fracture. Therefore, it is of great necessity to consider the potential damage of the thread during the preload process in FEA.

In the present paper, a tensile test and three-dimensional FEA were applied to a single bolt to reveal the mechanisms underlying the strength reduction and the fracture mode transition under preload and tensile load. Experimentally measured force–displacement curves were compared with the results from a FEA to correct the displacement. Lastly, the fracture mode is presented as a function of the stress distribution from the FEA and morphology of fracture analysis.

## 2. Experimental Study and Finite Element Analysis

**Figure 1:**
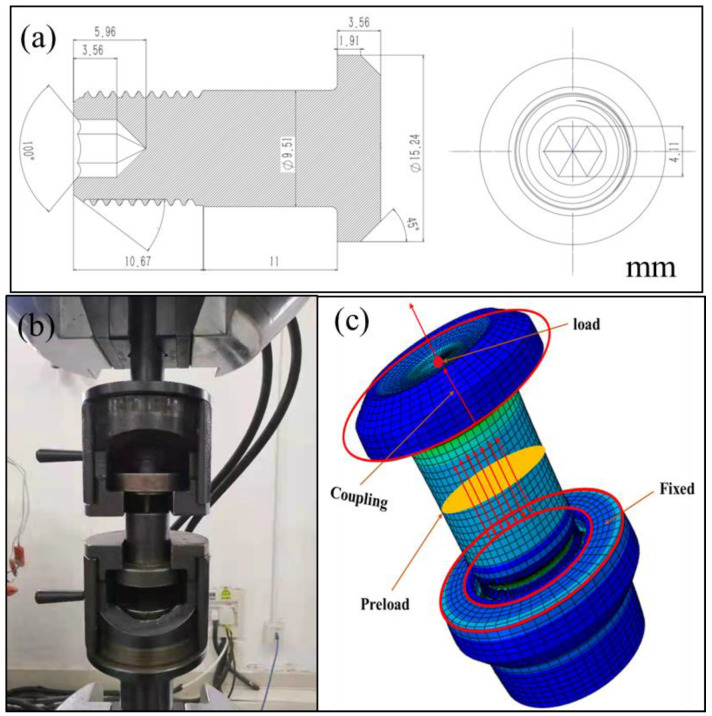
(**a**) Drawing of the bolt; (**b**) the test machine; (**c**) the basic FE model.

The specific details of the as-received TC4 bolt are shown in Figure 1. The standard composition and measured composition are listed in Table 1. The samples for microstructure characterization were machined from the cross-section. Firstly, samples were ground using SiC sandpaper down to 1400 grit, followed by polishing with diamond grinding paste to a mirror surface. Then, cleaning and erosion were carried out. The metallography of the as-received sample was observed using SEM.

The specimens for the tensile test were fixed by two fixture plates which were linked with a tensile testing machine, as shown in Figure 1b. The bolt was places across the hole in the upper plate, and the thread end was screwed into the threaded hole in the bottom plate. The plate specimens were subjected to a tensile test with a load rate of 875 N/s until ultimate fracture. Simultaneously, the load and displacement were recorded. It should be noted that the observed displacement was machine displacement, which included bolt displacement, clamp deformation, and the assembly clearance between the bot and clamp. For the fractured bolts, the fracture surfaces were investigated using an FEI Sirion IMP scanning electron microscope (SEM) system equipped with an energy-dispersive spectroscope (EDS).

The CAX4 element was used in ABAQUS to establish an axial symmetry model for the single high-lock bolt, as shown in Figure 1c. In order to obtain accurate results and improve the convergence rate, different mesh densities were applied to the shank and thread. To reveal the effect of mesh size, different meshes from 0.03 to 0.08 mm were used to build the thread; the corresponding meshes of the shank were 0.3 to 0.8 mm. Next, mesh sizes of 0.5 mm and 0.05 mm for the bolt shank and thread were selected to reveal the stress distribution. The transition zone between the shank and thread was established with a gradient mesh size.

The boundary conditions are shown in Figure 1c. The nut had five fixed displacement degrees, except for the displacement along the axis direction. One reference point was defined at the center of the axis of the bolt and coupled with the rigid area. A rigid constraint was imposed on the reference point of the bolt. To simulate the assembly situation, a preload of 28.9 kN was applied to the high lock bolt using the “bolt load” option in ABAQUS. The preload force was selected according to the assembly guidance of this bolt. FEA was carried out to study the displacement and stress distribution using a linear elastic constitutive mode. The total reaction force could be directly obtained from the reference point, and the imposed displacement was easily applied to simulate the tensile test. In the finite element model for the high-lock bolt, a surface-to-surface contact model between the screw pair was defined. The friction coefficient of the contact was 0.15. The density of TC4 was 4.5 g/cm^3^. The Young modulus was set as 110 GPa with a Poisson ratio of 0.34 according to [21].

## 3. Results and Discussion

### 3.1. Microstructure of As-Received Bolt

**Figure 2:**
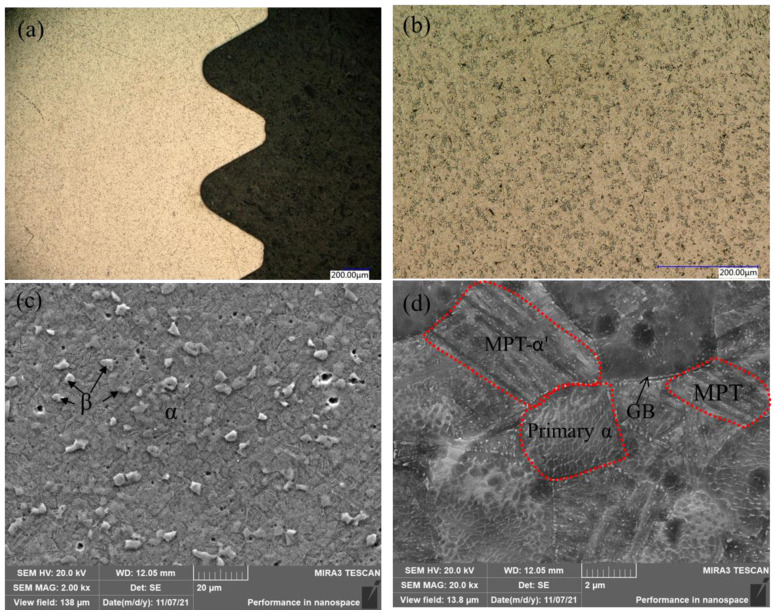
Microstructure of the bolt: (**a**,**b**) optical microscopy; (**c**,**d**) SEM.

The metallography of the as-received bolt was investigated by optical microscopy and scanning electron microscopy to observe the microstructure after processing, as shown in Figure 2. It is universally acknowledged that the original TC4 mainly consists of α phase with a dispersive distributed small precipitation β phase. The observed dark points in Figure 2a and the island-like phases in Figure 2b indicate the precipitated β phase. To further determine the size and detailed phase evolution, magnified graphs were evaluated using SEM, as shown in Figure 2c,d. The grain size of α phase was calculated to be about 7 μm, while the β phase accounted for about 14% of the surface. The observed α phase was larger than the reported value because of the rammer process, which resulted in obvious grain growth in the α phase [22]. In addition to the observed α and β phases, many small grains with lamellar structure can be seen in Figure 2d. According to the literature, the small grains should be the secondary α phase (α′) formed through the martensite phase transformation (MPT) of the equiaxed primary α phase [23]. The formation of the α′ phase in this work was attributed to rammer process-induced deformation. The α′ phase nucleates at the (primary α–prior β and prior β–prior β) grain boundaries and grows into β grains, resulting in a lamellar colony structure. The formation of a small α′ phase would further enhance the strength [24]. Therefore, the microstructure of the as-received bolt consisted of a high proportion of α phase, some β phase, and a small amount of α′ phase formed via martensite phase transformation during the rammer process.

### 3.2. Mechanical Properties

**Figure 3:**
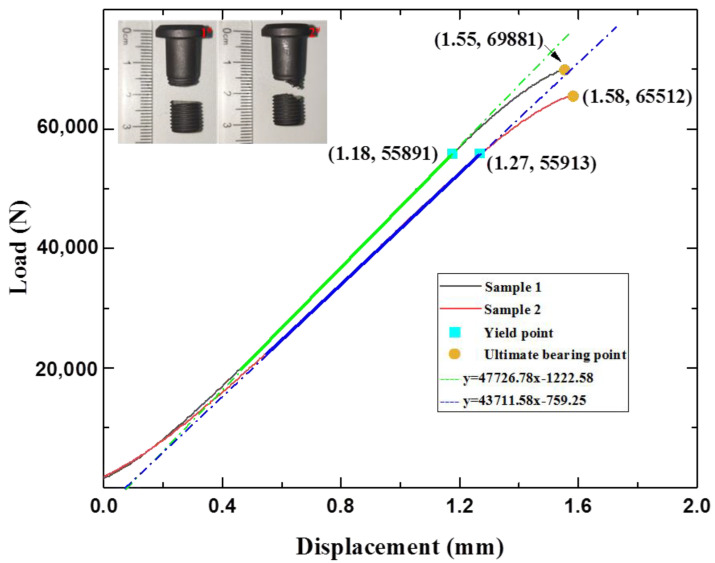
Load–displacement curves obtained by tensile experiments.

The load–displacement curves of the tensile samples are presented in Figure 3. The two curves revealed a similar increase pattern under tensile load, indicating the accuracy of the tested results. According to the standard file for mechanical testing of fasteners (ISO 898-1:2009), fitting formulas were developed on the basis of the line segment corresponding to elastic deformation of the bolt. The yield point and break point of sample 1 were obtained at displacements of 1.45 and 1.58 mm, while those of sample 2 were obtained at displacements of 1.42 and 1.55 mm. The slight differences in the obtained displacement (less than 0.5%) could be experimental errors due to clamping or machine recording. It should be noted that the observed difference in displacement was much larger than that in load between these two samples. The observed displacement (over 1.5 mm) referred to machine displacement, including bolt deformation, clamp deformation, and diminishment of the assembly clearance, which was much larger than the value of the TC4 standard tensile sample. The average correspondent loads for the yield point and break point were 55,900 N and 67,652 N, respectively. The errors of the critical load for yield point and break point were 0.1 kN and 2.2 kN, suggesting adequate repeatability of the tested samples. Furthermore, the yield stress and tensile stress calculated by dividing the load with the cross-sectional area were 0.906 and 0.952 GPa, respectively. The calculated tensile strength was slightly lower than the standard 1.1 GPa; the underlying reason was further explored as a function of stress distribution and fracture morphology. The fractured bolts are shown in the inset of Figure 3. It can be seen that the fracture occurred in the threaded section, especially the second thread. The calculated loaded area always changes under tensile load because of the spiral structure of the thread, which leads to uneven loading of the thread. To reveal the uneven loading, FEA was carried out for load area calculation and stress distribution, as described in the next section.

### 3.3. FEA

**Figure 4:**
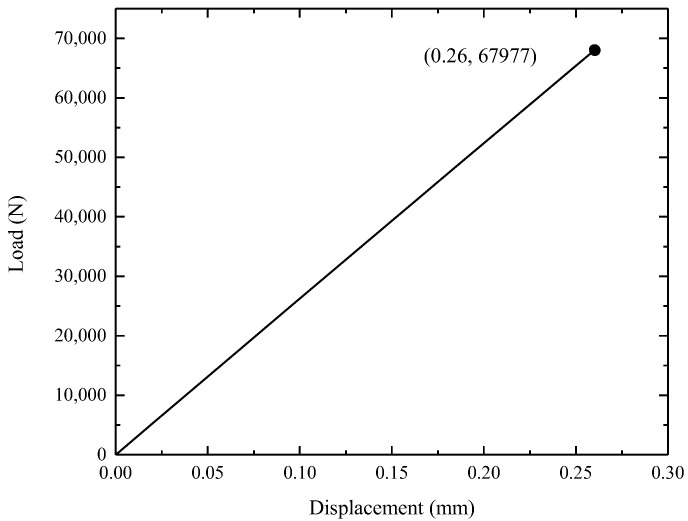
Load–displacement curve obtained by FEA.

The load–displacement curve according to FEA is presented in Figure 4. As the tensile load increased to 67.9 KN, a displacement of 0.26 mm was obtained from the simulated curve. The simulated displacement was much lower than the experimental displacement, indicating that the machine-recorded displacement was not only the bolt displacement.

**Figure 5:**
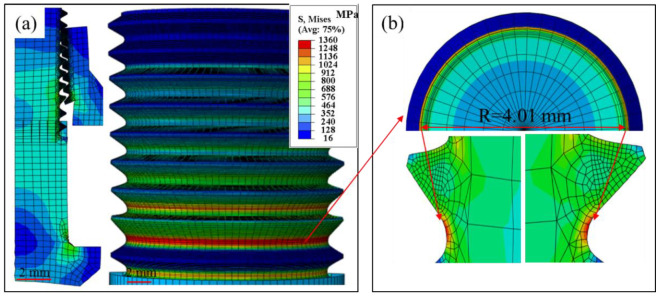
Stress distribution of the bolt under a preload, (**a**) stress nephogram of thread, (**b**) stress distribution of the thread root from cross section.

Furthermore, the detailed stress distributions induced by preload and tensile load are presented with stress nephograms. Under a preload, an obvious stress concentration could be observed in the roots of the threads, as shown in Figure 5a,b. The largest stress of 1.36 GPa was observed on the surface of the second thread when a 28.9 kN preload was applied. The concentrated stress in the root was larger than the reported tensile strength of 1.1 GPa, which would lead to strain hardening and even crack initiation on the thread surface [22]. According to the stress distribution, preloading induced stress centering on the area from the surface to the screw center. The area, which possessed strength over 1.1 GPa, was calculated to be 0.675 mm in depth from the thread top surface. This means that the loading area for tensile load was decreased to a circle with a diameter of 8.16 mm instead of the original 9.51 mm (the screw diameter) because of the initial cracks in these areas. Accordingly, the calculated tensile strength of sample 1 and sample 2 could be described by the formula, $\sigma =F/\pi r^{2}$, where *F* is the tensile load, and *r* is screw diameter. The tensile strength of the bolt screw was corrected to be 1.23 GPa and 1.29 GPa for sample 1 and sample 2, respectively.

**Figure 6:**
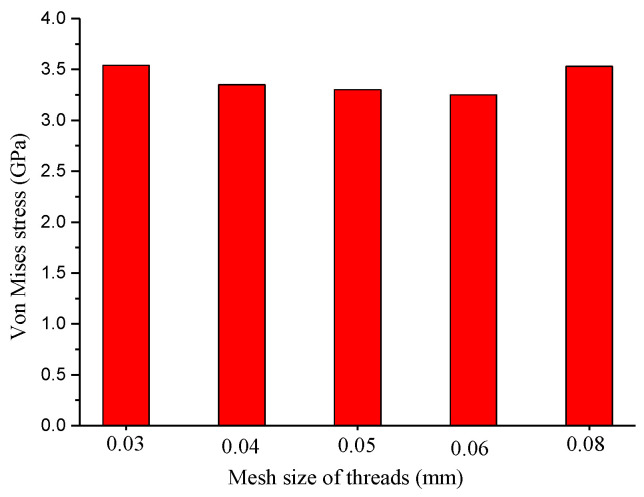
The Von Mises stress as a function of mesh size.

**Figure 7:**
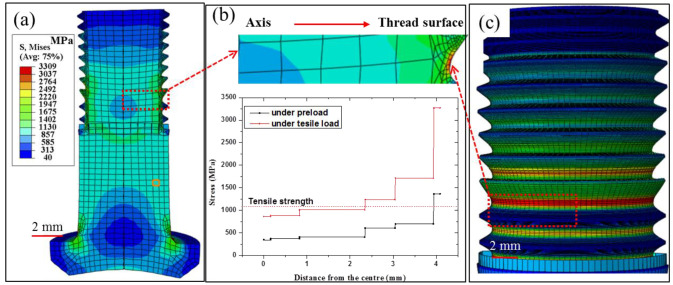
Von Mises stress distribution of the bolt under tensile load, (**a**,**c**) stress nephogram of thread, (**b**) stress distribution of the thread root from cross section.

As tensile load was applied to the bolt, the stress increased with the increase in load. To explore the effect of mesh size on the stress calculations, the maximum Von Mises stress as a function of mesh size is presented in Figure 6. As the mesh size of the thread increased from 0.03 to 0.05 and 0.08 mm, the maximum Von Mises stress first decreased from 3.54 to 3.30, and then increased to 3.53 GPa. The difference in maximum Von Mises stress induced by the mesh quality was about 6%, demonstrating that the computational results were independent of grid resolution. The stress distributions obtained from the models with 0.05 mm and 0.5 mm meshes are displayed in Figure 7. Figure 7 reveals that the principal stress was heterogeneously distributed and decreased from the screwing parts to the unscrewing parts. The heterogeneous force distribution should be related to the preload during assembly. The maximum stress was still observed in the second and third threads, consistent with abovementioned fracture location in Figure 3. Under tensile force, the second thread surface revealed a stress of 3.3 GPa. This stress was about three times larger than the measured tensile strength, which would lead to rapid cracking in thread. The stress distributions from the screw axis to the thread induced by preload and tensile load are presented in Figure 7b. The preload from the assembly drew a stress of about 1.13 GPa on the screw, resulting in strain hardening and initial cracking in the thread. As the tensile load was applied, the initial crack quickly propagated, and brittle fracture appeared in the strain hardening area of the thread. Then, these cracks propagated toward the screw center, which possessed excellent plastic deformation ability. To prove the validity of these findings, the fracture morphology of the tensile bolt is presented in the next section.

### 3.4. Fracture Morphology of the Tensile Bolt

**Figure 8:**
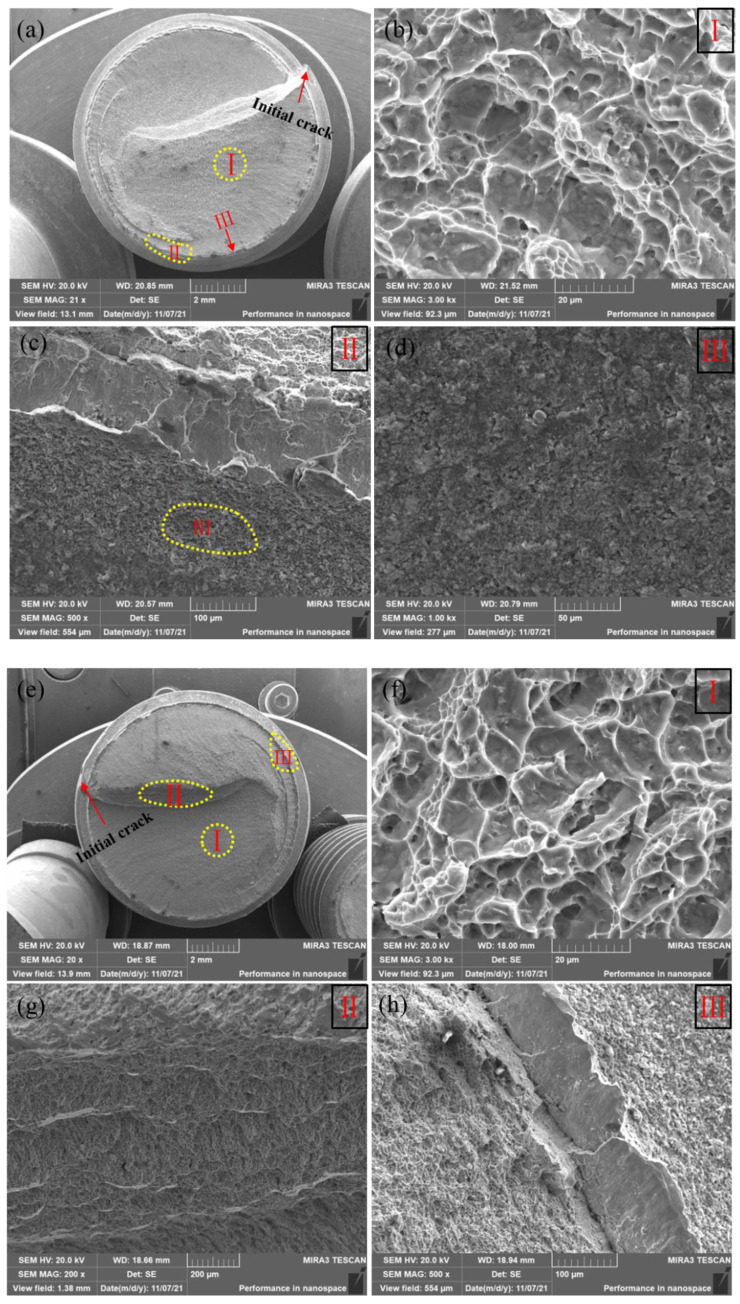
Fracture morphology of tensile samples: (**a**–**d**) for sample 1; (**e**–**h**) for sample 2.

To identify the fracture process and explore the fracture mode, fractography observations of the tensile tested samples were carried out using SEM, as presented in Figure 8. In the images with lower magnification, both fracture bolts revealed a large smooth region (marked as I) in the center and a small shear lip (marked as III) around the outside of the thread. An obvious step could be found in the center of the screw. The higher-magnification images of the smooth region and shear lip are also presented. The smooth regions of the two samples showed similar fractographs, presenting a large number of dimples (Figure 8b,f). These large dimples were formed through the growth of voids and deeply fractured portions, observed as a smooth region under higher magnification. The size of the dimples was calculated to be about 17 μm, indicating excellent resistance to deformation [25]. The excellent deformation capacity was related to the high 14% β phase in the samples, reported to be beneficial for ductile fracture [26]. However, fracture areas near the steps (marked as II and III) presented totally different morphologies with an obvious shear lip, as shown in Figure 8c,g. No dimple was observed. This is typical brittle feature, indicating the limited deformation ability of the thread. Thus, it could be concluded that the TC4 bolt after assembly revealed a mixed fracture mode. Brittle fracture was mainly distributed around the outside of the thread with a small initial crack retained, while ductile fracture occurred in the screw parts with many dimples distributed. The reason for the appearance of local brittle fracture regions is likely related to the strain hardening which emerged earlier in the roots of the threads because of the extrusion force during assembly [27,28]. In addition, the initial cracks in the external thread were arranged in a spiral ring because of the 4° lead angle of thread. Thus, a step was formed as initial cracks met each other after propagating from the outside to the screw center, as shown in Figure 7a and Figure 8a.

### 3.5. Fracture Mode of the Assembly Bolt under Tensile Load

Titanium alloy Ti6Al4V is a two-phase alloy which possesses good plasticity due to the spring back property and hcp crystal structure of the micro-constituent ‘α’ phase. For high-lock bolts, the fracture is always ductile fracture [29]. However, the fracture in this work revealed a hybrid regime. The reason for this hybrid fracture is discussed on the basis of experimental and FEA results.

**Figure 9:**
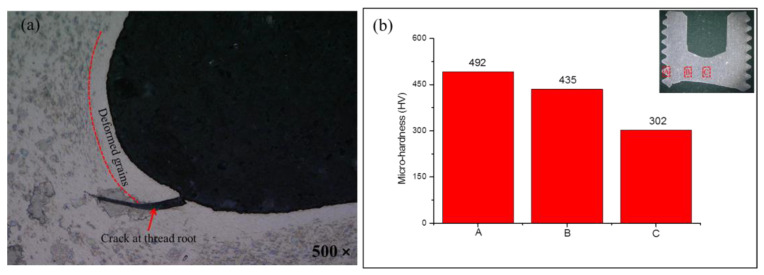
(**a**) Initial crack and (**b**) micro-hardness in marked areas of fracture bolt.

The fracture bolts suffer from both assembly and loading processes in turn. During the assembly process, a preload of 28.9 kN was applied on the bolt, which resulted in large compressive stress in the thread, as shown in the FEA results in Figure 5. The high compressive stress of ~1.3 GPa could provide a high pre-tightening force to the bolt against looseness, which also led to serious strain hardening in the thread. Strain hardening was identified by the higher hardness of 492 HV obtained around the thread compared with 302 HV in the screw center, as shown in Figure 9. This obvious strain hardening was reported to greatly compromise the deformation capacity of TC4 [30]. When tensile stress was applied to the bolt, the stress concentration of the thread roots was further intensified. The initial cracks on the thread surface, as shown in Figure 9a, quickly propagated and induced fracture in thread, as confirmed in Figure 8a,e. Because of the strain hardening induced by the preload, the fracture in the thread was brittle fracture with a shear slip formed on the outside of the thread (as shown in Figure 8). Then, the cracks propagated to the screw as the stress concentration induced by assembly and tensile loading exceeded the breaking strength [31]. The crack propagation led to a decrease in the load-bearing area of the bolt. Thus, the tensile load-bearing area of the bolt needs to be corrected by subtracting the crack propagation area in the thread [32]. Taking the effect of initial crack propagation from the strain hardening thread into consideration, the breaking strength of the assembly bolt could be calculated as shown below.

$$\sigma_{b}=\frac{F}{\left(S-\Delta S\right)}$$

where *F* is the maximum load, *S* is the cross-sectional area of the screw, and Δ*S* is the area of shear lip.

For the inner part of the screw, TC4 retained excellent toughness without a strain hardening effect. Thus, the fracture was ductile fracture with a large number of dimples, as shown in Figure 8b,f.

In conclusion, the fracture process of the assembly bolt could be described in three stages; firstly, the thread underwent a strain hardening process and initial crack generation under assembly; secondly, the initial crack in the strain hardening area led to brittle fracture under the combined tensile stress; thirdly, the screw broke because of the crack propagation toward the screw axis under the synergistic effects of tensile load and decreased cross-sectional area. Thus, the fracture of the bolt was a hybrid mode mainly consisting of ductile fracture in the screw and brittle fracture in the thread. The FEA and experimental results suggest that the preload of high-lock bolts should be controlled to prevent strain hardening and crack generation in the thread during assembly. Accurate preload control can be obtained using FEA, thus providing scientific guidance for the assembly of high-lock bolts to avoid assembly-induced fracture failure.

## 4. Conclusions

An experimental study and finite element analysis were carried out in this paper to explore the fracture mode of TC4 high-lock bolts under tensile load after assembly. The following conclusions could be drawn:

1. The TC4 bolt consisted of mainly α phase with some precipitated β phases. A small α′ phase with a lamellar structure was formed through martensite transformation during rammer process;
2. The tensile test indicated that the yield stress and elongation of the assembly bolt were 0.9 GPa and 1.2 mm, which were successfully used to verify the finite element model. The FEA results presented a Von Mises stress of 1.3 GPa on the thread root under preload, which induced obvious strain hardening on the thread surface. The tensile load brought about stress in the bolt exceeding its strength, which led to the final fracture.
3. The fracture mode of the assembly bolt was a hybrid mode consisting of brittle fracture and ductile fractures. Brittle fracture initially occurred in the thread and developed into ductile fracture in the screw. The initial brittle fracture was induced by strain hardening resulting from the preload and obvious shear lip, while the ductile fracture in the screw revealed a large number of dimples. The initial cracks in the thread also led to a step in the fracture cross-section.

The results successfully disclosed the fracture mode transition of assembly high-lock bolts under tensile load, and they provide a strategy to analyze the failure of in-service bolts. FEA can be applied as a strategy to calculate the preload during assembly to avoid strain hardening-induced fracture failures for high-lock bolts. Furthermore, the crack propagation issue should be further investigated using the finite element model to visualize the crack process, which would be helpful in explaining the fracture of bolts under the synergetic effects of preload from assembly and tensile load from service.

## Figures and Tables

**Table 1:** Composition of TC4.

Elements	Al	V	Ti	Fe	O	Si	C	N	H	Others
**Standard** **(%)**	5.5–6.75	3.5–4.5	rest	0.3	0.2	0.15	0.1	0.05	0.01	0.5
**Measured** **(%)**	6.27	3.96	89.77	-	-	-	-	-	-	-

## Data Availability

The data are available in a publicly accessible repository.
